# Exploring the physical activity of Iranian migrant women in the United Kingdom: a qualitative study

**DOI:** 10.1080/17482631.2021.1963111

**Published:** 2021-08-06

**Authors:** Nasrin Soltani, Julie Botticello, Paul Watts

**Affiliations:** a Independent Researcher, UK; b Department of Health Sport and Bioscience, University of East London, London, UK

**Keywords:** Physical activity, migration, Iranian migrant women, social practice theory, United Kingdom

## Abstract

**Purpose:**

This article explores the role migration has on the physical activity of Iranian migrant women living in the United Kingdom.

**Method:**

This qualitative study includes 22 first-generation Iranian migrant women, aged 24–64, residing in London. Data was collected through in-depth, semi-structured, individual interviews and was analyzed thematically.

**Results:**

The findings show that for those women from traditional backgrounds, migration corresponds with liberation from social and cultural pressures experienced in Iran and greater motivation to adopt a physically active lifestyle. However, for Iranian women who had arrived in the UK more recently and had a higher social standing in Iran, migration was associated with the loss of their careers, sources of income, and social networks. These issues were compounded by a lack of cohesion in Iranian migrant communities and poor access to local physical activity resources. This resulted in diminishing motivation and the subsequent de-prioritisation of the women’s physical activity, even though they had regularly engaged in physical activity in Iran.

**Conclusion:**

Despite migration leading to improving physical activity for some Iranian women, for others, migration leads to marginality in Britain. Local authorities and Iranian community organizations need to adopt innovative strategies to reach out to recent Iranian migrant women.

## Introduction

Concerns over the lack of physical activity among migrant communities in Western countries have been well documented (Fischbacher et al., 2004; Long et al., 2009; Williams et al., 2011). In the United Kingdom (UK), much research has focused on South Asian migrant groups (Babakus & Thompson, 2012; Daniel et al., 2013; Fischbacher et al., 2004), whereas smaller migrant communities remain under-researched (Gatineau & Mathrani, 2011). The Iranian community in the UK, with a population of approximately 70,000 (Office for National Statistics, 2016), has been described as invisible (Spellman, 2004). Although it is not very large, with the increasing number of asylum-seeking Iranians coming to the UK, the community continues to grow (Full Fact, 2016). The emphasis of this study is on Iranian migrant women in the UK, because their lifestyle practices, in particular their physical activity, have rarely been the focus of research.

Adults between 18 and 64 are recommended to perform at least 150 minutes of moderate-intensity or 75 minutes of vigorous intensity physical activity each week (World Health Organisation [WHO], 2020). The UK Chief Medical Officers add more detail and recommend that adults in this age group combine moderate-intensity and vigorous intensity physical activity together with resistance training two or more times a week. This should be spread throughout the week, by being active most days, and concentrated in bouts of 10 minutes or more per session (Davies et al., 2019). To obtain the health benefits of physical activity, adults are advised to increase their daily activities. This includes walking regularly, in particular brisk walking, cycling as transport, increasing activity levels while performing daily routines, taking regular breaks from sitting whether at home or work, walking up stairs and reducing TV or screen viewing time (National Institute for Health and Care Excellence, 2013). In spite of these guidelines, across the globe, many individuals do not engage in sufficient levels of physical activity for maintaining good health (Guthold et al., 2018).

Multiple interrelated personal, cultural, social, economic, environmental, community, and policy related factors influence the physical activity of individuals (Sallis et al., 2008). Social environments, such as family, friends and broader social networks play a significant role in the physical activity of migrant women. Since this group is likely to face loss of support from extended families, relatives and community networks, they may encounter more challenges for participation in leisure-time physical activity than women from the host society (Caperchione et al., 2011; Persson et al., 2014). These challenges were reflected in the focus group discussions of Middle Eastern migrant women, including Iranians, residing in Australia (Gholizadeh et al., 2011). The study participants were mostly from low socio-economic backgrounds. Their limited social support, poor English skills, lack of employment and financial insecurity resulted in excessive life pressures and diminished opportunities to engage in physical activity (Gholizadeh et al., 2011). In another Australian study, predominantly with highly educated Iranian migrants, stress at the initial stage of migration caused them to adopt a sedentary lifestyle, but upon assimilation to the new society, they were more motivated to participate in physical activity (Delavari et al., 2013). The female respondents identified other reasons for being more physically active in Australia, including the absence of Iranian government pressures on women’s dress code, in particular wearing hijab, and the lack of social pressures on maintaining a slender body shape (Delavari et al., 2013), a deeply held belief among Iranians in their home country (Abdollahi & Mann, 2001). Furthermore, the respondents, the majority of whom were medical doctors, asserted that the availability of mixed gender gyms, access to green spaces, and being able to observe people exercising in public venues inspired them to become more physically active themselves (Delavari et al., 2013). The contrasting views in these studies with Iranian participants in Australia, might be related to their sampling strategies, with each focusing on one socio-economic group, which resulted in definitive, yet polarized, portrayals of migration, rather than representing nuances within each group.

The cost of gyms is another well-documented obstacle to participation in leisure-time physical activity across migrant and host populations (Caperchione et al., 2011; Gholizadeh et al., 2011; Lawton et al., 2006; Persson et al., 2014). Migrant women, however, are more likely to be financially disadvantaged due to their higher unemployment rates (Gatineau & Mathrani, 2011). This would limit their abilities to join a gym, since for some, their husbands are the main breadwinners and decision makers in the household, and as such, might not find the cost of gym justifiable or necessary (Gholizadeh et al., 2011).

The physical environment can also affect physical activity engagement. Stokols (1992) theorizes that the environment can operate as a facilitator of physical activity, exemplified by the proximity of parks or fitness facilities to one’s home. Residents living in more walkable areas are less likely to be obese (Giles-Corti et al., 2013), whereas those living in geographically unsafe areas are less likely to engage in physical activity (Stokols, 1992). Moreover, weather adversity is another deterrent to participation in physical activity for migrants, including those not used to cold weather in the host country (Daniel et al., 2013). While several studies conducted in Western countries (Caperchione et al., 2011; Gholizadeh et al., 2011; Grace et al., 2008; Lawton et al., 2006; Persson et al., 2014) have reported that Muslim women who followed Islamic requirements for modest dress face great difficulties accessing gender segregated sports facilities, the above studies highlight that the challenges for physical activity engagement may also be related to diminished social networks and barriers in the physical environment.

## Theoretical framework

Social practice theory (Shove et al., 2012) provides the theoretical foundation for this study. Practices are defined as the interdependent relations between elements including “forms of mental activities, ‘things’ and their use, a background knowledge in the form of understanding, know-how, states of emotion and motivational knowledge” (Reckwitz, 2002, p. 249). In an attempt to simplify the definition, Shove et al. (2012) proposed that practices, such as cycling to work, walking as a means of commuting, and eating dinner in the family context, depend on the ongoing integration of three key elements: materials, competences, and meanings. Materials encompass objects, tools, hardware, consumer goods, infrastructures and the body itself (Shove & Walker, 2007). Competences involves practical knowledge or know-how of a situation, knowing where, when and how to perform a practice. Meanings refer to the customs, norms, and embodied understandings of the social and cultural significance of the practice (Shove et al., 2012, p. 23). Practices should be seen through a framework of the three elements. Shove et al. (2012) explains how the changing relationships between the three elements lead to formation, transformation and continuation of the practice. For instance, a break in the links between the defining elements of any one practice can cause aspects to disappear, with the changes integrated into the new version of that practice. This highlights that practices are not static, but emerge, transform, persist and disappear.

The other central point about theories of practice is the importance of individuals as hosts or “carriers” who “carry out” and sustain practices (Reckwitz, 2002, p. 256). Specifically, social practice theory seeks to understand the initial encounter of individuals with practices and how practices attract, recruit and expand through social networks and communities (Blue et al., 2016). The theory takes into account the variations within any given practice and how, where, how consistently, for how long, and on what scale it is produced. The continuation of a practice depends on changing populations being more and less committed as carriers or practitioners (Shove et al., 2012, p. 66).

Pred (1981) focuses on the life paths of individuals, the practices and “dominant projects” they carry. From Pred’s point of view, “individuals” daily paths are structured by projects and priorities that have consequences for the accumulation of competences and dispositions’ (Pred, 1981, p. 14). For adults, he emphasizes that having children, changing occupations and migrating each have a significant impact in setting their dominant projects; this has implications for people’s time, energy and efforts. Pred’s analysis suggests that “not all practices are equal; instead lives revolve around a handful of dominant projects” (Pred, 1981, p. 16). Dominant projects give rise to certain priorities taking up the individuals’ time and attention in certain directions and not others. This has consequences for the skills and expectations they develop. As a result, “individuals” lives are woven into the reproduction of dominant societal institutions’ (Pred, 1980, p. 16). Borrowing from Pred’s discussion, Shove et al. (2012) suggest that practices compete with each other for time and effort. By applying social practice theory and its key elements, this article aims to understand the impact of Iranian women’s migration and resettlement on willingness or ability to engage in physical activity, with respect to their motivation and the impact of cultural norms. The article also explores the previous experiences of physical activity engagement and how one’s ability to participate in physical activity is related to competences, social networks and access to environmental, material and financial resources. The study participants were further encouraged to identify challenges for engaging in physical activity and to suggest ways that could ameliorate these situations.

## Methods

In response to calls for greater understanding of migrants’ physical activity through their subjective accounts (Blue et al., 2016; O’Driscoll et al., 2013), this research employs a qualitative research methodology. In keeping with social practice theory, it aimed to capture the experiences and views of Iranian women about physical activity, and used semi-structured individual interviews as the method of data collection. The study inclusion criteria were: being female, having been born in Iran to Iranian parents, having moved to the UK at age 17 or older, and currently being aged between 24 and 64. This was to “focus on prime-age adults since young adults typically have not established their post-adolescence residence” (Wen & Maloney, 2014, p. 125) and own practices arising. Choosing 17 as the minimum age of migration was to ensure that the participants had completed their childhood in Iran, offering them the ability to compare and reflect on their experiences in Iran and those in the UK. The study site was limited to London due to the high concentration of Iranians in the capital (BBC, 2005; Communities and Local Government, 2009; Harbottle, 2000).

## Sampling and recruitment

The recruitment process began upon receiving Ethical approval from the University of East London research ethics committee (UREC) and involved three phases. NS, responsible for data collection, initially connected to participants via her existing networks. This then gave her wider access to the Iranian community through snowball sampling, by building up the sample through the networks of the researcher and other participants (Patton, 2015). This phase garnered five participants in their 30s, 40s and 50s, all of whom were residents of the London borough of Barnet. In the second phase, the aim was to diversify the study sample by applying maximum variation sampling, or heterogeneity (Patton, 2015). During this phase, NS visited the most active Iranian charities, a number of Iranian schools and mosques, and attended various events in the community at which she distributed flyers inviting participation in the study. This phase lasted three months and garnered a further six participants in their 30s, 40s and 50s. The third phase was focussed on filling in gaps in the study sample by targeting Iranian women in their 20s and 60s, those with young children, and practicing Muslims. This phase resulted in gaining ten more participants. Follow up interviews were also conducted with six participants to offer more detail on their initial accounts. All participants have been given a pseudonym to protect their identity. Table I provides details of the participants’ demographics.

**Table I.** Demographic profile of the participants

Name	Age	Marital status	No children	Legal status	Education	Occupation	Number years in UK	Annual family income
Pantea	24	Single	None	Student visa	Student	Student	4	No data
Atousa	28	Married	One	British	Bachelor’s degree	Housewife	11	£30–45 K
Aida	29	Married	One	Spouse visa	Bachelor’s degree	Housewife	3	£15–29 K
Ellahe	30	Single	None	British	Master’s degree	Charity worker	12	£15–29 K
Farzane	34	Separated	One	Refugee	High school diploma	Apprentice	<1	<£15 K
Zahra	35	Married	Two	Refugee	Associate degree	Apprentice	6	No data
Farnaz	37	Married	One	British	Bachelor’s degree	Housewife	9	£15–29 K
Ghazal	37	Divorced	One	Refugee	High school diploma	Apprentice	5	<£15 K
Shiva	37	Single	None	Refugee	Masters’ student	Private tutor	11	<£15 K
Nastaran	38	Married	Two	Refugee	Bachelor’s degree	Apprentice	4	£15–29 K
Shayesteh	38	Married	Three	British	Bachelor’s degree	Housewife	12	£30–45 K
Parvin	44	Married	Two	Refugee/British	High school diploma	Apprentice	11	<£15 K
Fatemeh	46	Married	Three	British	College leaver	Housewife	28	<£15 K
Nazanin	46	Married	Two	Refugee/British	Bachelor’s degree	Apprentice	6	<£15 K
Samira	46	Married	Two	British	Bachelor’s degree	Voluntary worker	13	No data
Taraneh	50	Married	Three	Refugee/British	High school diploma	Housewife	15	£15–29 K
Mahin	52	Married	Four	Asylum seeker	Middle school	Housewife	4	<£15 K
Mahvash	52	Married	Three	Refugee/British	Bachelor’s degree	Recent graduate	10	£15–29 K
Shahnaz	55	Single	None	British	Bachelor’s degree	Office worker	38	£15–29 K
Tahereh	55	Widow/ divorced	None	British	Bachelor’s degree	Teacher	18	£15–29 K
Mitra	59	Married	One	British	Bachelor’s degree	Accountant	40	>£45 K
Rohi	64	Married	One	Refugee/British	Bachelor’s degree	Retired nurse	15	£15–29 K

## Data collection and interview guide

The interview guide was informed by physical activity literature and social practice theory. It included open-ended questions that aimed to explore the meanings attached to physical activity and the contexts in which physical activity was carried out. In keeping with social practice theory, the research aimed to understand whether migration disrupted, continued, or initiated physical activity. The questions were meant to identify the impacts of their social networks, employment statuses and financial statuses on physical activity engagement. The respondents’ evaluation of their neighbourhood characteristics, as representations of their physical environment or materials, both in Iran and the UK, and how these influenced their participation in physical activity, was also explored. The guide included questions on identifying the obstacles to physical activity and asked for recommendations to improve physical activity among women (see Table II). The participants were free to suggest a convenient public location and the language (English or Farsi) in which to be interviewed. Of the 22 initial interviews, 17 were conducted face-to-face within the participants’ locality at various cafés, schools, charities and parks. Five were carried out by phone at the participants’ request. The average length of interview was 56 minutes. The data collection ended with 31 interviews: 22 initial interviews, and nine follow-up interviews.

**Table II.** Interview guide

1)	How did you find this move?/How did you find living here?
2)	How do you spend your time?
4)	How do you describe physical activity?
5)	Thinking about physical activity, in the last week what kind of physical activity, in what locality, for how long and what time of the day did you perform the physical activity?
6)	How does your physical activity here compare with what you would do back home in Iran? If you think of the time you were in your homeland, what changes in your physical activity level have you noticed?
7)	What reasons would you or someone from your community identify for not participating in physical activity? (Probing for barriers such as cost of gyms, language barrier, lack of social network etc.)
8)	How did you find your neighbourhood? (Probing for facilitators and barriers to their physical activity.)
9)	Could you suggest some solutions for improving physical activity levels of women in your neighbourhood?

## Data analysis

All interviews were audio-recorded and transcribed verbatim; those conducted in Farsi (Persian) were also translated into English by NS. All the transcripts were imported to NVivo 11, which assisted in storing, coding and managing the data. The data was analysed using thematic analysis, using the six-stage protocol recommended by Braun and Clarke (2006, pp. 16–20). Thematic analysis fits with different theoretical frameworks, including social practice theory, as it aims to identify both patterns of meaning and provides an analysis of participants’ subjective meanings (Flick, 2014). Braun and Clarke (2006) six stages include familiarization, generating initial codes, searching for themes, integrating them into broader themes, defining themes, and producing the report. To establish credibility, sustained engagement in the field was maintained over a year-long period. To increase the reliability of the data (Lincoln & Guba, 1985), nine follow-up interviews were conducted with six participants who were willing to add more detail to their accounts. Further, four participants agreed to read their interview transcripts and provide feedback, affirming the transferability of data between the research participants and the researchers. NS, who was responsible for the data collection, would regularly share the transcripts, field notes, codes and themes with JB and PW. In their monthly discussions, JB and PW would similarly interpret the data, which process resulted in refining the themes into four main categories. These were: “Experiences of physical activity in Iran”, “Motivation, time management and liberation from socio-cultural pressures”, “Migration and stress”, and “Navigating the new society, challenges, resources and preferences”.

## Results

### Experiences of physical activity in Iran

The experience of physical activity in Iran was linked with the women’s upbringing, social context and norms, their family attitudes to gender, their social class and the time they departed Iran. Among those very well-settled in the UK is Taraneh, aged 52. She has been living in the UK for 15 years, and recalled her experience of physical activity in Iran. Coming from a liberal family background, she noted “Overall, I had been a sporty person from high school. I was playing in the school’s volleyball team; I was good at all types of sports.” Although these themes were echoed by those belonging to conservative families, Fatemeh, 46, who has been living in the UK for nearly three decades, reflected on her childhood and adolescence experiences of physical activity where she felt enormous pressures from her family, limiting her physical activity and movements, as she explained:

I didn’t have much freedom as a woman in my country because of my family upbringing. I wasn’t even allowed, you know, to go to high school [myself]; my mother just took me to high school … Not all the family are like that but my family, my father was like that … We have those constraints … I couldn’t even ride the bike, because my brother had a bike and I wanted to ride it but they didn’t allow me.

Fatemeh, in her narrative demonstrated the magnitude of the constraints her parents imposed on her. While the gender differences in permitting who could try different types of physical activity in Fatemeh’s family is evident, she tended to limit the negative personal experience of physical activity to her own traditional family, rather than generalizing it to all Iranian women. Yet, some long standing Iranian migrants in this study shared similar experiences. When considering recent migrants, this quote was repeatedly mentioned: “I was much more physically active in Iran.” The narratives of several recent migrants who had 10 or fewer years of residence in the UK, signified their familiarity with formal and/or informal types of physical activity prior to their migration. This is not only a representation of the change in the country’s social and cultural norms but is also related to the women’s educational attainment and skills that enabled them to hold a job and lead a financially independent life whilst in Iran. Zahra, 35, with six years residence in the UK, reflected on her life experience as a wedding organizer and artist: “When I was in Iran, I was so active all day long.” Her career was not only a reason to keep her active in the community, but also provided her with financial stability to afford the cost of gyms. As she added later: “I used to go swimming, also going to the gym a lot in Iran.” There were other participants who were not necessarily financially privileged, yet they were inspired by immediate family members or wider social networks to regularly participate in physical activity. Iran’s sunny weather, and the availability of neighbourhood parks, proved to provide invaluable opportunities for physical activity. Mahin, 52, a housewife, currently an asylum seeker with four years of residence in the UK, talked about the influence of her daughter on her physical activity:

Where I used to live, we would wake up early, going to the local park with my daughter, even if I was lazy, my daughter would wake me up and take me there from 7 to 9 … there was equipment which we would use for half an hour, using the bicycle, some machine for strengthening wrists, and dumbbells …

As the above could be interpreted, one solution to overcome laziness is the peer pressure or encouragement of social networks, that can influence the types of physical activity undertaken and the use the exercise equipment in neighbourhood parks. Similarly, other participants such as Aida, 28, an immigrant with 3 1/2 years of residence in the UK, highlighted the Iran’s sunny weather and low rainfalls facilitated her, along with her families and friends, to arrange various outings in nature: “Picnicking, which was not only sitting; we would go for a walk, playing with family, mountain climbing with friends every Friday.” The frequent references to performing physical activity in the neighbourhood and beyond suggest that for staying physically active, a woman would not necessarily need to join a gym. Her social network could offer ideas and bring people together in organizing different forms of physical activity. Yet, going to gyms for middle-class participants was essential. Nazanin, 46, a recent refugee with 6 years of residence in the UK, explained why she was more physically active in Iran due to the educational qualifications she held and her professional jobs in Iran:

My physical activity level was much more than here maybe four times more … when I was in my country, I had reached a financial stability … therefore I could deal with other matters, one was paying attention to my body and fitness a great deal … in Iran, we would pay attention to our appearance, especially we would wear ‘Manto’. Well, with Manto, which was our outdoor outfit … if I were fat, it wouldn’t look nice on my body, so it was embarrassing.

Although Nazanin referred to her privileges, such as financial stability that assisted her in covering the costs of gyms, achieving a slim body shape was to avoid embarrassment of deviating from the body shape set by the social group she belonged to, a symbol of her social class and status. Hence, working out at a gym would assure her of maintaining this standard and remaining in her social group. This is despite the fact that women in Iran must wear a Manto, an outfit similar to a raincoat for covering the body, when appearing in public.

Through the lens of social practice theory, we can extrapolate that social and cultural norms as the representation of meanings plays an integral role in allowing or prohibiting Iranian women’s participation in physical activity. This subsequently influences their competences in practising physical activity whether in the past or current era. Those women belonging to traditional family backgrounds experienced various degrees of oppression beginning from their childhood through adolescence and possibly later in adulthood, whereas women from more liberal families had opportunities to play in their neighbourhood and at school. Although the cultural constraints on Iranian women’s physical activity still exist (Mirsafian et al., 2014) there is a visible shift in the account of recent Iranian migrant women who demonstrated varying experiences of engaging in physical activity. This is regardless of their legal, educational, and financial statuses or whether they are asylum seekers, refugees, immigrants, housewives, employed, working class or middle class. The drastic shift in Iran’s cultural attitudes towards women’s social and physical activities suggest that culture norms are susceptible to change, such as when the Iranian government improved infrastructure, or materials, through building public and local parks as well as private gyms at a national scale. With the abundance of sport facilities free of charge in public parks, combined with the availability of private gyms for those with sources of income, more women could engage in physical activity, demonstrating that meanings and materials enhance competences of Iranian women in physical activity participation. The nuance of social and cultural norms in contemporary Iranian society would not only see participation in physical activity for some women a stigma, but also created new meanings for participation in physical activity in which other women tended to inspire their family members and/or wider social networks to engage in a variety of leisure time physical activities. Some appeared to be engaging in physical activity for fun, health, or a way of socializing. Some were obliged to practice it to ensure they fitted into the standards of their social group. Regardless of their reasons, Shove et al. (2012) stress that through rapid interactions new members take up a practice and create a larger community of fellow practitioners.

### Motivation, time management and liberation from socio-cultural pressures

Those participants who felt themselves to have settled well tended to see women as independent adults, and believed that motivation, autonomy, and time management were key factors in engaging in physical activity in the UK. Autonomy in this context refers to the women’s ability to have control over their own behaviours, daily schedules and make wise decisions (Beltrán-Carrillo et al., 2019). Some women such as Mahvash, 50, a former refugee with ten years of residence in the UK, described her own busy daily schedule as a mother of three children, to demonstrate the feasibility of participation in physical activity for a woman:

Because at the time, when I went to the gym, to be honest, I tried to do my cooking, and cleaning quickly, even I would go once a day to primary school, a teaching assistant voluntary job, you know. I do that even when I go to the gym, I try to come back home by walking, not by bus, because I feel OK, if I come back I can, you know, do more burning, more calories. And my size was 8–10, you can’t believe that, but after that you can see, if you want something, you can do it.

Mahvash, by exemplifying her own daily routines, signified the importance of autonomy and motivation in ability to multi-task and fulfil multiple daily responsibilities that enabled her reward of achieving a slim body shape. Some women referred to many positive aspects of UK society in removing the barriers they experienced in Iran. Shayesteh, 38, an immigrant, with twelve years of residence in the UK, and a mother of three, strongly believed that she was leading a more physically active life in the UK:

Here I’m more active, because there [are] less limitations … If you want to take your children to the swimming pool, for instance, could I take my son to the pool if I were in Iran? No … here, if I want to run, regardless of my body shape, no one looks at me to say “Oh look at her she’s so fat and even can’t walk but she’s running.” But if it was in Iran, you get hundreds of verbal abuses. Secondly, where would you go for a run? But here, it’s not important, no one judges you, here you feel relaxed; you can wear whatever you like.

By comparing two countries’ social policies, cultures and environments, Shayesteh identified a variety of reasons why physical activity to easier to take up in the UK. Safe streets, lack of street harassment, and judgemental people, freedom of dress code and UK mixed gender gyms provide her with the peace of mind to maintain physical activity alongside her children of opposite sex in the UK. Resettlement in the UK, for those who experienced extreme oppressive environment in Iran, was equivalent to opening new horizons, including their spouses endorsing their physical activity. Fatemeh, who previously spoke of her life in Iran, explained how she became physically active in the UK:

Actually, my husband made a small gym at home. But still I’d like to go outside but when the weather is not good, I try to use the gym …, my husband helped me to ride a bike and had a helmet and everything, a flasher thing for safety (laughs).

Feeling empowered by the support of her Iranian husband who equipped the home with sports devices and mentored her, she also distinguished between indoor and outdoor physical activity. The preference for outdoor walking was frequently mentioned by other participants for enjoying beautiful scenery, releasing their tension, and boosting mental well-being. Shayesteh and Fatemeh’s comments both highlighted the positive role of migration through changes in cultural and social norms by removing barriers to initiation of physical activity for those who grew up in oppressive environments. This represents the role of “meanings” in initiation or boosting physical activity of women. As with the previous theme, access to suitable materials, such as mixed gender gyms and the support of a spouse in providing equipment and mentoring, appear to be instrumental in enhancing the women’s competences in performing physical activity with or without family members.

### Migration and stress

Contrary to those who felt liberated, migration, for some of the recent respondents was linked to sustaining varying degrees of stressful life events. For some such as Nazanin, 46, a married woman with two children, one of whom was very small and constantly ill, stress was at the family level. Living in the UK for six years, she recalled her memories in the first year of her arrival:

When I immigrated to the UK, I lost my middle class standing and financial stability. I arrived in a country where there were more important matters for me to be concerned with, such as finding a suitable place to live and a decent job so that I can bring my children up in a suitable environment. … I stopped thinking about myself and taking care of my health … . paying attention to health was not my priority at all or was at the bottom of my priority list … . this affected my health and will be continuously going to do so.

Migration for Nazanin was associated with enormous losses; social class, financial status, and accommodation amongst others. For a migrant woman with a family, other necessities were prioritized. By separating her “self” from the list of her everyday agenda, she could focus on more fundamental issues required for her family survival. She had arrived in a country where she was not competent in speaking the language of the majority; therefore, her qualifications and job experiences were not recognized. This caused her financial hardship, loss of position and status, with deeper detrimental effects on her sense of identity and belonging to UK society. Her story demonstrates the direct link between life pressures, mental health and physical health. In these circumstances physical activity became a luxury, whereas her life stability in Iran would grant her the possibility to practice physical activity regularly. Ghazal, 37, a single refugee who has lived in the UK for five years, identified social isolation and the psychological impacts of migration as the underlying reasons for becoming sedentary in the UK:

My physical activity level is very low … I have nowhere to go, mostly staying at home … I’ve got depression, during the day, if I feel OK, I go for a walk for an hour, otherwise just sleeping … I have never been as inactive as I am right now … (In Iran) I would go for a walk in the evenings with my mum and sister … (here) I wanna go for a walk but I can’t find a walking buddy to go out with …

Ghazal’s story was multidimensional, in which she had to handle various life adversities given her unfamiliarity with the environment, legal system, poor language skills and with no access to a supportive social network who could guide her, comfort her daily stresses and provide company. By stating “nowhere to go” Ghazal might not necessarily mean the lack of familiarity with her surroundings but the absence of inspiring people who would motivate her to go out. This was compounded by the crippling impact of depression, meaning she would only manage occasional physical activity. Britain liberated Shayesteh, but for Nazanin and Ghazal it was lack of social support and being faced with mounting daily pressures that had adverse effects on their mental well-being and diminished their motivation to participate in physical activity. Ghazal explicitly identified not finding company for walking as one of the underlying causes of a lack of motivation to participate in physical activity. This might, to some extent, be related to the lack of cohesion in the Iranian community in Britain (Spellman 2004) and inaccessibility of a reliable social network for recent migrant women (Communities and Local Government, 2009).

Although the refugee women in this study suffered more psychological trauma, there was no shortage of expressions of feeling down, depressed and lonely among those who had migrated without seeking asylum. Yet, some would purposefully avoid communicating with other fellow Iranians. Aida, from a socially well-connected family was an example, earlier mentioned enjoying family outings and mountain climbing with her friends in Iran, and like other recent migrants, acknowledged leading a much more physically active life in Iran. Recalling the early months of her arrival, she spoke of feeling so lonely and experiencing depression, yet she would still prefer disengaging with fellow Iranians: “I didn’t find Iranians interesting, because they have made a small Iran with different cultures and groups … which is much worse than home, with all competitions, jealousy, gossips going on.” From Aida’s point of view, the community of Iranians in the UK mirrored similar features to that of Iran, filled with rumours and social comparisons, and for these reasons she would rather distance herself from them. Similarly, others described Iran’s culture as “weak”, and found staying away from other Iranians a way to a successful resettlement. The issue of lack of cohesion within the community of Iranians in the UK was highlighted by even very well-connected study respondents. Mahvash spoke of having regular gatherings with other Iranian and non-Iranian families in her house, and meeting with Iranian women’s friends on a weekly basis. She, however, acknowledged the negative attitudes of Iranians to one another:

You know, it is only us, Iranians, who dislike one another. I have seen this right from the beginning of my arrival in this country. I was advised by other Iranians not to enroll my children in the schools with a high number of Iranians … but you don’t see this attitude in other minority ethnic groups, for instance, in Afghans.

Mahvash singled out Iranians with a negative approach to other Iranians as one of the fundamental issues in the community. She also envied other migrant communities such as Afghans who were frequently described by other interviewees as “nice” people who make up a cohesive community. Mitra, 59, who came to this country as a student and has been living in the UK for four decades, spoke of being encouraged by her neighbour to join the rambling society. Becoming a regular rambler, she wondered why she has not succeeded to inspire other Iranians: “I have not managed to get some of my Iranian friends [to] walk.” Recent migrants had no means of getting to know the well-settled physically active Iranians whom they could befriend and receive support from in navigating and overcoming the everyday challenges in the new society, as well as stay physically active. The dynamics within the Iranian community in Britain and the issue of distrust of other Iranians played a large part in diminishing the women’s motivation to participate in physical activity. As revealed in the stories of recent migrants, prior to migration, most of them were leading active lifestyles and were inspired by people in their social networks. But, in the absence of such community in the UK, they had to handle mounting life pressures that would take up their mental and physical energy. Hence, they felt no motivation, nor would attach any positive meanings, to performing physical activity.

### Navigating the new society, challenges, resources and preferences

There were ample references, from nineteen participants, to the positive characteristics of their neighbourhood such as safety, aesthetics, and access to the green spaces that all contributed to their daily walks to work, their children’s school, and local facilities. Aida, a first time mother, highlighted the positive role of smooth pavements in enabling her to walk frequently with her child in a pushchair in the community, something that was not widely available in Iran: “I don’t see any barriers to physical activity, given the pavements are smooth that allow me to use a pushchair, except the weather.” Speaking of the negative impact of windy and wet British weather for Iranians coming from a dry to semi-dry climate (Weatheronline, n.d.), several women brought up the importance of accessing gyms, described as enclosed spaces to engage in physical activity, as a solution to the UK weather adversity and also a social hub to expand their social network. Farnaz, 37, who came to the UK with a spouse visa, felt very fortunate accessing a local discounted gym that brought her social benefits: “I found some [like-minded] Iranian friends, you know, you see more and more. If you stay at home, just it’s you.” The value of working outside home was frequently highlighted by other women. Only five participants were economically active (Table I), so given the women’s lower socio-economic status, access to low-cost neighbourhood gyms were key to participating in different types of physical activity. Nonetheless, not all had the privileges of accessing such spaces and some respondents specifically highlighted this issue as a major challenge. Fatemeh, who despite identifying herself a physically active woman, joined a gym for its therapeutic benefits, however, this was a discontinued practice:

I used to go to Pilates as well for my, um, joint pain …, and because I have got problem with my bladder and doing some exercise on those muscles. It helps you for, to strengthen your muscle as well which it cost me £10 per each hour and I couldn’t carry on. You know those things which you wanna do it, you wanna be happy, stay healthy but the money restricts you.

For Fatemeh, gyms not only boosted her physical health but also promoted her mental well-being, yet her financial constraints deprived her from such privilege. It was not only the cost of gyms, religious Iranian women participants appeared to face other challenges. Shayesteh’s and others’ accounts endorsed UK mainstream mixed gender swimming pools that enabled them to enjoy swimming with other family members, regardless of their sexes. This, however, was identified as an obstacle to three practising Muslims respondents. Despite all having the experience of joining mainstream mixed gender sports facilities and using exercise machines that demonstrated their interest in integrating into the British society, this practice had limits since neither felt comfortable joining the UK mixed gender swimming pools. The preference for swimming was echoed by all in this group; they independently identified many challenges of joining a gender segregated swimming pool, as Zahra described:

Swimming is good for me. I mean, every time I go my knees get better for a few days. I feel good in general from my emotional state, … but well, that one is so far away so my husband should spend a day to drive us off there, then pick us up.

Despite the health and well-being outcomes of swimming, inaccessibility of a local gender segregated swimming pool and the travelling hassles resulted in either intermittent participation or abandoning the practice altogether for almost all practising Muslim participants. Locating suitable physical activity resources was another issue that highlights the information seeking strategies of Iranian migrant women respondents. Seeking help from health professionals, such as GPs, for a variety of concerns was frequently mentioned by the interviewees. Nastaran, 37, a refugee who had lived in the UK for four years, believed her professional career in a prestigious company in Iran, would never leave her any free time to engage in physical activity. She, however, acknowledged that her migration facilitated her initial engagement with formal physical activity. She cheerfully spoke of a visit to the GP who referred her to “exercise on prescription” as a solution to her back pain: “GP sent me a free three months, so [I became] a little bit became familiar [with] what is a gym, what I can do … . at the moment, it’s like I became a member.” The information seeking of other respondents appeared to be limited to either word of mouth or use of flyers. Four respondents learned about their neighbourhood resources through word of mouth when communicating with fellow Iranians, such as via language schools, Persian schools, colleges and mosques.

In addition to identifying barriers to physical activity, the participants were encouraged to suggest ways of promoting physical activity for women. Several respondents believed that accessing a local low-cost gym, and for the religious women, culturally appropriate swimming pools, were key in enhancing participation in physical activity. A number of recent migrants also wished to access free group physical activity in their locality, information about which they hoped to receive through flyers. Ghazal spoke of participating in a free instructor-led walking group, having been informed of this initiative through flyers distributed in her college. She reflected on her experience:

I also joined a walking group in Croydon, It was very good. It was just for six weeks and was advertised by Kingston University, once a week two hours walking … It improved my English, I learned about the course that I’m currently taking. … besides I met new people.

In the above, Ghazal, demonstrated her desire for group physical activity as her willingness to integrate into British society. Although it was a short-lived programme, it benefited her occupationally, and socially. Nazanin, another recent refugee, requested group physical activity in places of worship:

In all areas, thank God there are lots of vacant halls at the churches or other public places and nobody uses them, so they can recruit a sports instructor, and distribute leaflets notifying the local residents, for instance, on Thursdays, at 6 pm. All women can get together here some days, but it must be free of charge (laughs) to motivate women to physical activity. Because if the council wants to improve women’s health (laughs) … Women tend to prioritize other needs over their own health, although they are not aware they must pay the price of ignoring their health needs in the future.

Nazanin’s request to participate in women’s group physical activity might not necessarily be a religiously orientated interest, since during the interview, she noted using mainstream swimming pools with her sons. Her interest in women’s group activity might be a way to expand her social network. She also highlighted the self-imposed financial limitations by prioritizing her children’s needs over her own physical activity. Nazanin’s lack of priority for spending money on gyms could be a reflection of various post-migration losses she previously noted. Nonetheless, her laughs implied the conflicts between her knowledge of the health benefits of physical activity and her lack of action. Nazanin recommended group physical activity in her local area, guided by a sports instructor with emphasis on free initiatives that reflect the significant changes in her life circumstances brought about by her migration. In the UK, she was unable to hold anything near to the professional positions she maintained in Iran with its associated disposable income. Furthermore, she suggested that in the context of migration and an absence of a social network, which tended to judge others based on body shape, she could no longer see the necessity of participating in physical activity to achieve that ideal. Further, paid physical activity programmes caused financial hardship on the already overstrained family budget. Speaking of participation in local group physical activity, neither of the recent Iranian migrant women had knowledge of their local council’s physical activity initiatives. This perhaps explains, to some extent, lack of competency among the study participants in identifying the local resources, despite being available in many languages on the local councils’ website. The local council’s magazine in English, however, was not helpful for recent migrants who might not be competent in reading English. With regards to the contribution of social practice theory, this theme highlights the major role of accessing suitable materials such as money, low-cost gyms, and for some, gender segregated swimming pools as well as having skills to identify various local physical activity resources and activities. These themes represent competences, in enhancing motivation and capability of Iranian migrant women to participate in physical activity.

## Discussion

To our knowledge, this is the first study to explore the role of migration in the physical activity of Iranian migrant women in the United Kingdom. This article has presented both overarching and nuanced themes on participating in physical activity in the UK, drawn from the reflections and experiences in Iran and Britain by Iranian migrant women, with analysis informed by social practice theory and its key elements of materials, competences, and meanings. The research aimed to understand how migration impacted on various aspects of physical activity including Iranian migrant women’s motivation to engage in physical activity and the impact of social and cultural norms, as well as women’s representations of the “meanings” of physical activity for them. The article explored the role of financial status and access to physical activity resources as manifestations of materials, and the influence of previous experiences in physical activity participation, together with other changes impacting on their competences to perform physical activity. The application of social practice theory revealed that the interconnections between materials, competences and meanings have led different outcomes for those from traditional backgrounds compared with those who newly migrated, on initiating, continuing or disrupting participation in physical activity. Those from traditional backgrounds felt their migration liberated them from the social and cultural pressures imposed by their families and Iranian society, which enabled them to initiate or improve their physical activity. In relation to the initiation and improvements in their practice of physical activity, a key aspect highlighted by the Iranian migrant women in this study was gaining autonomy over their daily schedules by setting some time aside for participation in physical activity. This represented both meanings of physical activity and competences to achieve it. Combined with materials, in the form of available and accessible infrastructure, including smooth pavements, low cost, local and mixed-gender gyms or home gyms, a further enhancement of their competences in swimming, cycling, and regular walking, was evident.

The testimony of those from conservative family backgrounds who grew up in oppressive environments revealed that migration was a transformative life experience for developing interest and competence in keeping a physically active lifestyle. This was due to accessing mixed-gender sports facilities, in particular swimming pools that enabled them to swim in the company of the family members of the opposite sex. This is in line with the accounts of middle-class Iranian migrants in Australia (Delavari et al., 2013). That the women who had experienced negative social and cultural pressures in Iran abandoned these norms in the UK suggests Iranians’ individualistic tendencies (Malek, 2015) and a desire to integrate into British society (Communities and Local Government, 2009). Further, the women’s spouses played a fundamental role in promoting the wives’ physical activity through verbal encouragement, which represented positive meanings becoming attached to physical activity. The husbands combined this with practical support, by mentoring their wives on how to ride a bike, driving their wives and children to swimming pools, and providing materials, such as home gym equipment, that collectively contributed to the women’s competences and skills in performing physical activity.

The sense of freedom from cultural norms and support from family led to the respondents constructing new perspectives on their gender and enhanced their beliefs that a woman, wife, and mother could be a confident and competent decision maker in managing her time. This finding around freedom and support also challenges the established discourse identifying that lack of previous experience of physical activity to be partially responsible for the low participation in physical activity among minority ethnic women in Britain (Grace et al., 2008; Jepson et al., 2008; Koshoedo et al., 2015). Further nuanced findings from this study pertained to the perception and experiences of practising Muslim Iranian women who did not refrain from attending mixed-gender gyms as long as they could modify their dress to comfortably be in these spaces. Previous studies suggested that practicing Muslim women would not feel comfortable to join mixed-gender swimming pools (Gholizadeh et al., 2011; Jepson et al., 2008; Koshoedo et al., 2015). That the London study participants regularly or intermittently attended sports facilities and used exercise machines demonstrates their willingness to integrate into British society, whilst adhering to religious norms. However, swimming remained a challenge due to the uneven distribution of gender-segregated swimming pools in different neighbourhoods (Long et al., 2009) and the challenges with travelling long distances resulted either in intermittent practice or abandonment of swimming, something many would have comfortably accessed and enjoyed in Iran. This demonstrates the integral role of the availability of suitable resources or materials for continuity in physical activity, and how the break in accessing such material resources can cause discontinuity in practice, even for those competent in the practice, prior to their migration.

Social practice theory also helped to understand the experiences of engagement in physical activity by the respondents who had migrated more recently. In their accounts, these women provided abundant examples of performing physical activity in Iran in their communities, in part due to their higher social status in Iran. This signifies the impact of community and group membership in stimulating interactions with one another, thus becoming “carriers” or “practitioners” (Crossley, 2006) who boost one another’s motivation to participate in physical activity. Nonetheless, in the context of migration, many respondents spoke of experiencing stress and psychological pressure, especially, at the early stages of migration, which negatively affected their mental well-being. This was compounded by a lack of cohesion in the community, manifested by a sense of suspicion in fellow Iranians. This distrust, frequently voiced by some study participants, resulted in their diminished motivation to participate in physical activity. The fragmented Iranian community in Britain is a phenomenon stretching back many decades (Spellman, 2004); it continues to marginalize recent Iranian migrants and has implications for their social mobility (Malek, 2015). In support of Malek’s argument, this research found that the fragmented Iranian community in London negatively impacted on new arrivals. For example, for those Iranian respondents who at the point of arrival lacked English language skills were deprived from receiving guidance from well-established Iranians who could have assisted them in navigating the new society and strengthened their coping strategies when facing the demands of life in the new society. The psychological effects of facing mounting daily pressures in the absence of supportive networks resulted in the women’s low motivation to participate in physical activity, whether in the short or long term.

This research also revealed the implications of migration for some recent migrants who had been participating in physical activity regularly in Iran but became far less physically active once in Britain. This is also reflective of social practice theory, as a break in the link between materials and access to services and resources diminished their motivation and therefore their competences in performing physical activity. Most of the recent migrant women, prior to their migration, had held a job or had had a supportive social network, reflective of a their previous middle-class status; once in Britain, due to language barriers and loss of social networks, finding suitable employment became almost impossible. This demonstrates the effect of downward social mobility, in the loss of social networks, careers and financial security. These constraints particularly impinged on the migrant women with children, as they could no longer justify the cost of gyms, whilst they would have enjoyed this in Iran, due to their higher status there. On the one hand, this inhibits women’s mobility and participation in physical activities among their fellow migrants in the country; on the other hand, it can also compel women to also seek other more welcoming community among local residents and even migrants for other communities in their search of finding communities in the UK. It may be due to this conundrum around lack of financial and social resources akin to what they had in Iran, combined with a desire to connect with a community, even if not an Iranian one, that some recent migrants were keen to join in free group physical activities. In terms of learning about these activities, this group also preferred to be informed of local initiatives through flyers; but local councils tend to rely on their website for disseminating their resources. Even though the local councils provided information in a variety of languages, the participants were not aware of the multilingual resources, nor could comfortably read the councils’ widely distributed newsletter in English. This suggests that the materials did not seek to engage with intended recipients and this impacted both on Iranian women’s competencies through not being able to practice physical activity, but more so, in the meaning of participation, that is through being connected and belonging.

Members of minority ethnic communities are poorly represented at decision-making levels and are excluded or encounter a range of barriers to sports participation (Koshoedo et al., 2015). It could be argued that poor participation of minority ethnic individuals in physical activity might partly be related to the issue of dissemination of information in minority communities. Long et al. (2009), in their research with Black, Asian and Minority Ethnic (BAME) communities stressed that more important than provision is communication. If information does not reach the intended recipient, or cannot be understood, due to not being translated, then it does not serve its purpose. Swinney and Horne (2005) argue that local authorities need to apply bottom-up approaches when working with minority individuals and communities, so that they are involved throughout the process. This is not just about provision of services, but is also about engaging with new groups, understanding their needs, and working together to meet them. A lack of community cohesion (Spellman, 2004) and an absence of representatives from the Iranian community (Communities and Local Government, 2009) have created fundamental obstacles for Iranians to raise their voices to engage with the local authorities. Moreover, there are so far limited partnerships between Iranian charities, schools, mosques and the local authority in designing cost-effective health programmes. However, as seen in this study, there is a willingness to engage in physical activity and to be part of supportive networks, from whatever community is willing to offer these. As seen in this study, social networks and the meanings derived from belonging are the primary vehicles for bringing people together for participation in physical activity. Migrant mobility in physical activity can be understood as their movement towards belonging in a new society. While the materials and the competencies are significant for accessing and maintaining physical activity levels, it is social networks and a sense of belonging which can enable Iranian migrant women to persist, emerge, and transform their practices anew as they make their way forward in their new lives in the UK.

## Strengths and limitations

As with other qualitative studies, the aim is not to generalize the findings to the population (Sofie Possmark et al., 2019), yet the diverse study sample of women from a wide age range, immigration history, family size, income level, educational and financial statuses yielded rich and in-depth subjective accounts of Iranian migrant women in the UK as evidence of trustworthiness of the study findings. The first author, being an Iranian migrant woman herself, wanted to avoid reproducing biases in her data collection. Therefore, throughout this process, JB and PW guided her to engage with a wider community, to recruit people from diverse backgrounds, and to cover a spectrum of the population, based on age, education, financial status, and class. The collective teamwork in constructing the interview guide, the decision to conduct follow-up interviews with some participants, and the choice to gather transcription feedback from some participants were also used to avoid biases. The contribution of all authors in data analysis was another measure for avoiding biases and gaining a nuanced interpretation of the findings (Delavari et al., 2013; Persson et al., 2014; Gholizadeh et al., 2011).

## Concluding remarks and recommendations

This exploratory study revealed that migration liberated some Iranian migrants from the social and cultural pressures experienced in Iran. This enabled them to initiate or improve their physical activity and adopt more physically active lifestyles. On the other hand, for more recent and middle-class and skilled women, migration was a cause for downward social mobility compounded by lack of cohesion in the community of Iranians in the UK. It, consequently, diminished the women’s knowledge of and access to the resources and support network which reduced their motivation to participate in physical activity. To increase migrant women’s participation in physical activity, it is recommended that women have access to local low-cost gyms and the provision of female only swimming sessions within the proximity of those with religious orientation is being increased. Future research should focus on designing interventions to determine the impact of peer support or peer led walking groups on enhancing the physical activity of Iranian migrants and their mental well-being. We also recommend that local authorities need to take a more proactive role in reaching the new migrant women from the early stage of transitions in familiarizing them with a new society. This can be achieved by providing them translated resources and linking them with women’s support groups, local active charities, libraries and social events. Future research should also involve various stakeholders such as local authorities, representatives from Iranian charities, mosques, schools, and institutions as well as lay and professional Iranian migrant women in identifying cost-effective and culturally appropriate health promotion programmes.
